# Improvement of the Durability of Recycled Masonry Aggregate Concrete

**DOI:** 10.3390/ma13235486

**Published:** 2020-12-01

**Authors:** Tereza Pavlů, Kristina Fořtová, Jakub Řepka, Diana Mariaková, Jiří Pazderka

**Affiliations:** 1 Department of Building Structures, Faculty of Civil Engineering of Technical University in Prague, Thakurova 7, 160 00 Prague, Czech Republic; jakub.repka@cvut.cz (J.Ř.); diana.mariakova@fsv.cvut.cz (D.M.); jiri.pazderka@fsv.cvut.cz (J.P.); 2 Research Team Architecture and the Environment, University Centre for Energy Efficient Buildings of Technical University in Prague, Trinecka 1024, 273 43 Bustehrad, Czech Republic; kristina.fortova@cvut.cz

**Keywords:** recycled masonry aggregate concrete, crystalline admixture, durability of concrete

## Abstract

The use of recycled masonry aggregate for concrete is mostly limited by the worse properties in comparison with natural aggregate. For these reasons it is necessary to find ways to improve the quality of recycled masonry aggregate concrete and make it more durable. One possibility is utilization of crystalline admixture which was verified in this study by laboratory measurements of key material properties and durability. The positive influence of mineral admixture was proved for freeze-thaw resistance. The positive impact to carbonation resistance was not unambiguous. In conclusion, the laboratory evaluation shows how to improve the durability of recycled masonry aggregate concrete, however, it is necessary to investigate more about this topic.

## 1. Introduction

The main advantages and disadvantages related to recycled aggregate (RA) used as aggregate for concrete are generally known and have been described many times. On the one hand, the replacement of natural aggregate (NA) leads to the reduction of primary source consumption. On the other hand, the utilization of recycled aggregate mostly negatively affects the properties of concrete, especially its water absorption, mechanical properties and durability. For this reason, the utilization of RA from construction and demolition waste (CDW) as aggregate for concrete has limited structural applications worldwide. Furthermore, the possible utilization of RA depends on its quality, composition, and properties. Generally, there are three basic types of RA known: recycled concrete aggregate (RCA), masonry aggregate (RMA), and mixed recycled aggregate (MRA) [1,2]. Each type of aggregate has its own specifications and limits of utilization.

The RCA mostly contains natural aggregate with attached mortar, unbound natural aggregate, and cement paste. The attached mortar causes two main problems related with RCA utilization as aggregate for concrete: (1) transition zones between old and new mortar which negatively influence the mechanical properties of recycled aggregate concrete (RAC) [3,4,5,6]; (2) the higher porosity and water absorption of RCA than the companion NA. The water absorbability of RCA, which ranges up to 15% [2], is necessary to know due to the mixture design for further workability of the concrete mix. These aspects lead to the higher water absorption, decline of freeze-thaw resistance and reduction in the carbonation resistance of recycled aggregate concrete (RAC) [7,8,9]. Generally, the replacement rate up to 30% of RCA has no significant influence on total porosity and compressive strength of RAC. When the replacement rate is higher than 30% of RCA in the mixture, the total porosity increases and compressive strength decreases significantly [5].

The freeze-thaw resistance of concrete mostly defines the possible future utilization of concrete structural elements in the exposition environment. As expected, the freeze-thaw resistance of RAC decreases with the increasing replacement ratio of NA by RA in the concrete mixture [10], as a result of the increasing porosity of concrete containing RCA [11]. Moreover, it is linearly correlated with its water absorption capacity [10]. In general, the freeze-thaw resistance of concrete is evaluated by comparison of several physical properties and mechanical properties before and after a defined number of freezing and thawing cycles [12,13]. During freezing, the water expands throughout the pores and makes pressure inside the concrete, which could lead to local cracks. Furthermore, the freeze-thaw resistance is also influenced by a water-cement (w/c) ratio, where increasing w/c ratio leads to decreasing freeze-thaw resistance due to the higher amount of mixing water.

The knowledge of carbonation resistance of concrete is essential due to the future utilization of reinforced concrete structural elements in which the corrosion of reinforcement would be affected. The reinforcing bars are endangered by corrosion when the passive coating is destroyed by carbonation and chloride ingress. Corrosion of steel bars starts earlier in RAC than in NAC and depends on the amount of RA in the RAC. Furthermore, the steel corrosion is also influenced by the electrical resistivity of concrete which decreases with the increasing replacement rate of aggregate and porosity of concrete. Carbonation of concrete, which can be described as a physical-chemical process, is influenced by the permeability of concrete, its moisture content, cement content and water/cement ratio, mineral additions, aggregate type, porosity and the environment’s CO_2_ content, relative humidity and temperature [9,14]. Generally, according to the previous studies [1,14,15,16,17,18,19,20,21,22,23,24] the carbonation depth of RAC also increases with an increasing replacement level of aggregate in concrete. The significant decline of carbonation resistance is shown for concrete with a higher replacement rate than 30% [17,23]. This is caused by the higher water absorption of RA than NA, which leads to a greater porosity of RAC than NAC the same w/c ratio [9,13]. In previous studies [9,25], the greater negative influence of the fine fraction of RA in comparison with the coarse fraction of RA was evaluated. This result was probably caused by the higher water absorption of fine RCA in comparison with coarse RCA, which leads to worse permeability of RAC. The carbonation depth of RAC tested during time periods is approximately two times higher than the carbonation depth of NAC [26]. Generally, it could be said that the results of limitation of the RCA utilization as aggregate for concrete in the case of carbonation resistance corresponds with the limitation in general, whereas the maximal replacement rate is defined as 30% for the coarse fraction (4–8 mm and 8–16 mm) and 20% for the fine fraction (0–4 mm) of RCA. On the contrary, the opposite result was also observed in which the RAC carbonation depth decreased. In this case, the replacement ratio of NA by RCA was greater than 70% and RCA had about 40% of adhered mortar. This phenomenon could be caused by the cement content in the adhered mortar, which could slow down the carbonation rate [27].

In spite of the negative aspects of worse carbonation resistance of RAC, there is one positive point of view. Concrete in general absorbs CO_2_ from the atmosphere. RAC with higher porosity and consequently greater carbonation depth absorbs more CO_2_ then conventional concrete, which brings environmental benefits [28,29].

The masonry aggregate (RMA), with a high content of waste masonry such as red bricks, calcium silicate bricks, ceramic blocks, aerated concrete, plaster, etc., and mixed recycled aggregate (MRA) which combines RMA and RCA, has not found satisfactory use yet. According to the Czech standards [30], the RA with a lower content of waste concrete than 90% is not efficiently used as aggregate for concrete, due to its negative impact on mechanical properties and durability. The properties of RMA, such as porosity, water absorption, density, and resistance—for instance, resistance to wear, abrasion resistance, or freeze (thaw resistance), etc. are different in comparison with NA, which depends on the composition which is connected with the recycling process and consequently influences the properties of recycled masonry aggregate concrete (RMAC) [31,32,33,34,35,36,37,38,39,40,41,42,43,44,45,46,47,48,49,50,51,52,53,54,55,56].

In the case of RMA, higher porosity and water absorption are caused by porous materials contained in RMA, such as red bricks, ceramic blocks, aerated concrete, and concrete particles with adhered mortar. It follows that the consequences of high porosity and water absorption of RMAC are slightly different than that of RAC; however, the impact on the mechanical properties and durability is similar. For better properties of fresh and hardened concrete, the additional water compensating water absorbability of RMA needs to be added to the concrete mixture during mixing [43] or the RMA must be soaked in water for 24 h before mixing [49]. Generally, the mechanical properties and durability of recycled aggregate concrete decrease with an increasing replacement rate of aggregate in concrete by RMA [43,49,57]. A maximal replacement rate without significant decline of mechanical properties of RMAC comparable with NAC has been found in 15% coarse RMA. On the contrary, the mechanical properties of RMAC with 100% replacement rate decreases up to 65% [49]. The partial replacement of natural sand by fine RMA has had no significant impact on the compressive strength of recycled aggregate concrete, probably due to the high amount of fine particles and silica and alumina contents in crushed bricks, which could lead to pozzolanic reactions [57]. Moreover, it was found that the carbonation depth of concrete with RMA is greater in comparison with RCA for the same replacement ratio of aggregate. Furthermore, the carbonation depth of concrete with MRA, the combination of RMA and RCA, increases with increasing amount of RMA in the mixture. It follows that the masonry content worsens the carbonation resistance [9].

Durability of concrete is the ability of concrete to resist various types of effects during its service period of exposure to its surrounding environment. The durability of RMAC is usually weaker than NAC and RAC due to its higher porosity and water absorption. In previous studies, the possibilities of durability improvement were verified. At first, there were few verified possible ways to improve the freeze-thaw resistance of RAC. When RCA is presoaked during the two-stage mixing approach [58] and the water absorption of RCA is compensated (the use of additional water resulting from the absorbability of RCA), the concrete mix achieves a higher compressive strength and durability [59,60,61], due to the water contained in the porous RA affecting the internal curing effect, in which the water is gradually released for further cement hydration [61,62,63]. Furthermore, the addition of a suitable amount of fly ash or metakaolin [64,65,66] to the concrete mixture is due to the ability of the mineral admixtures to react with Ca(OH)_2_ to form an additional C–S–H gel to increase the density and strength of concrete. Secondly, it was found [67] that the carbonation depth of RAC at early ages could be positively influenced by water-reducing superplasticizers. However, the effectivity of this solution decreases with time. The use of superplasticizers leads to the crystal growing, which causes the structure of concrete to be denser. Moreover, the other way to improve the carbonation resistance of RAC is the addition of mineral admixtures [13] such as high volumes of fly ash, due to its ability to fill the pores, which consequently improves the microstructure. [68]. On the contrary, the utilization the mineral additions as a partial replacement of cement causes a lowering of pH of the concrete and leads to worse carbonation resistance [9]. Finally, the other way to reduce the carbonation depth is the lowering of the w/c ratio [9].

The main aim of this study is the improvement of the RMAC mixture durability by the crystalline admixture. This approach is completely new due to the fact that there are no studies where the durability properties of RMAC concrete were improved and furthermore, no studies where the properties of concrete with RA are improved by crystalline admixture. The combination of these two topics is the main aim of this study. The verification of improvement in RMAC properties was proven by the laboratory measurements of the physical and mechanical properties and durability performance, especially freeze-thaw resistance and carbonation resistance.

## 2. Materials and Methods

In total, 8 concrete mixtures were prepared and tested to verify the possibility of improvement of the RMAC properties by crystalline admixture. Crystalline admixtures (CA) are permeability reducing admixtures, which are inherently hydrophilic due to their easy reaction with water. The chemical reaction between the crystalline admixture with cement and water leads to the increasing density of calcium silicate hydrate (CSH) and resistance of water penetration. The proper function of the waterproofing effect of crystalline material in concrete is achieved by the reaction of various chemical components necessary when the porous system of concrete reaches a sufficient level of moisture [69]. It was found that the crystalline admixture could improve the durability of reinforced concrete [70]. For example, the depth of penetration could be reduced by almost 50% in selected cases [71]. The previous experimental measurements verify the positive impact of crystalline admixture on the durability of concrete [72,73,74,75].

There were two types of NAC mixtures—one was a reference mixture with a natural aggregate without crystalline admixture, and the other mixtures contained two types of RMA as a full replacement of NA and different amounts of crystalline admixture. The durability properties were tested in two steps. The first four mixtures (NAC 1 and RMACs 1) were tested at age 28 days. The second four mixtures (NAC 2 and RMACs 2) were tested at age 60 days to show the influence of the crystalline admixture over time [74,76].

### 2.1. Recycled Aggregate

In the previous studies, it was found that the dry density of coarse RMA ranges between 1800 and 2700 kg/m^3^ and fine RMA between 2000 and 2500 kg/m3, which is generally lower than natural gravel and sand [32,40,43,49,52,53]. The range of water absorption of coarse RMA has been established from 10% to 19%, which is up to 25 times higher than natural gravel [32,40,49,52], and fine RMA from 12% to 15%, which is more than 10 times higher than natural sand [43,52,57].

**Figure 1:**
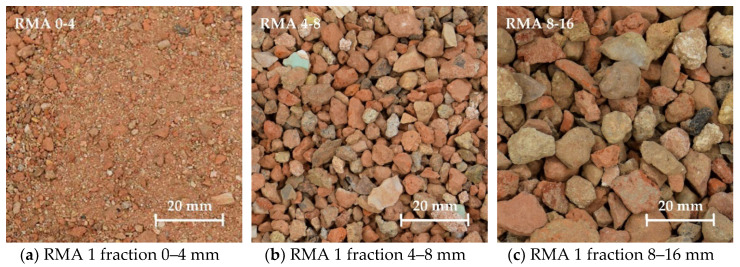
Recycled masonry aggregate (RMA 1).

**Figure 2:**
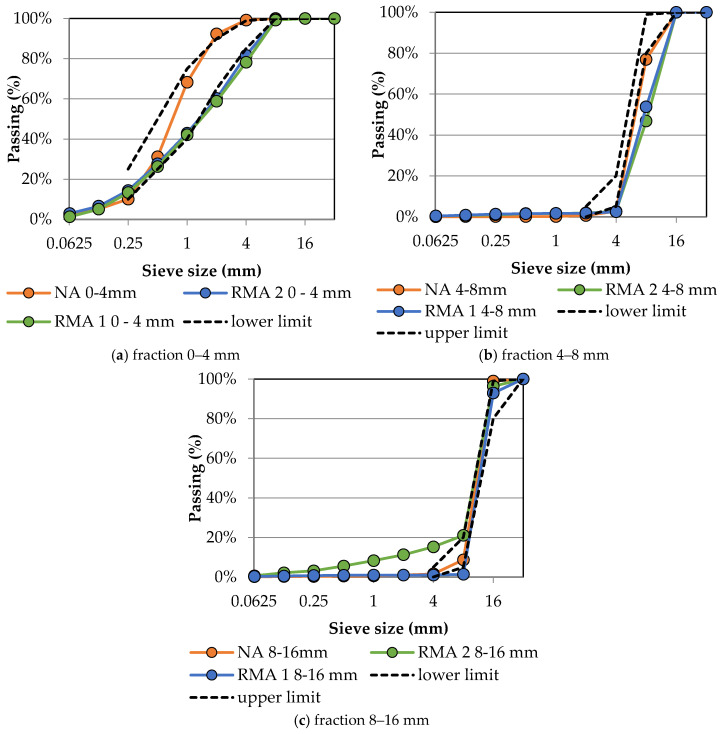
Sieving curves for natural aggregate, recycled masonry aggregate with limits defined in the standard [77] used in concrete mixtures.

In this study, one type of NA and two types of RMA were used. Both types of RMA were prepared from construction and demolition waste by a Czech recycling center, which were separated into fractions of 0–4, 4–8, and 8–16 mm (see Figure 1). Both types of RMA contained more than 70% of the waste masonry (red brick, aerated concrete, and plaster). Furthermore, the waste concrete and unbound aggregates were contained. All tested properties of RMA differed from NA, especially the water absorption capacity, which was more than ten times higher and ranged from 7.8 to 12.4% for coarse fraction and was 3.7 and 13.3 for fine fraction of RMA. This evaluation shows slightly lower water absorption of RMA in comparison with results of previous studies [32,40,43,49,52,57]. The dry density of RMA was lower in comparison with NA with a decline up to 25% which corresponds with the results of previous studies [32,40,43,49,52,53]. Furthermore, the RMA contains more fine particles and has different granulometry in comparison with NA and does not meet the requirements in Standard [77] (see Figure 2). Therefore, the basic properties of aggregates (see Table 1) are presented to show the differences in the materials used for the preparation of the concrete mixtures.

Selected properties of RMA were tested according to the requirements of Czech European standards [77]. The properties with the highest impact to the recipe design were tested. The basic properties of RMA are shown in Table 2 and the granulometry is shown in graphs in Figure 2.

### 2.2. Recycled Aggregate Concrete Mixtures

The laboratory measurements were carried out on eight concrete mixtures with the same exposition class XC1, effective w/c ratio 0.65, and amount of cement CEM I 42.5 R 260 kg/m^3^. The mixtures were optimized using the Bolomey particle size distribution curve. The additional water was calculated for RMAC mixtures according to the water absorption capacity of RMA and moreover the higher effective w/c ratio 0.65 positively influenced the effect of the crystalline admixture. Two mixtures of conventional concrete (NAC 1 C0 and NAC 2 C0) of strength class C25/30 only with NA of particle size up to 16 mm and without crystalline admixture was manufactured as a reference to compare with the other mixtures in which NA was fully replaced by RMA. Two mixtures of RMA concrete (RMAC 1 C0 and RMAC 2 C0) were prepared without the crystalline admixture. In the other four mixtures, the crystalline admixture was added to improve the properties of RMAC. Mixture RMAC C1 contains 1.5% (of cement weight) of crystalline admixture and mixture RMAC C3 contains 3% (of cement weight) of crystalline admixture (see Table 2).

Physical and mechanical properties and durability were tested at age 28 (NAC 1 and RMAC 1) and 60 days (NAC 2 and RMAC 2) according to valid Czech standards. Samples of dimensions 100 × 100 × 400 mm^3^, 150 × 150 × 150 mm^3^, and 100 × 100 × 100 mm^3^ were used for testing.

### 2.3. Evaluation Methodology

The mechanical properties were tested on Controls MCC8 50-C8422/M (Controls Group, Milan, Italy) according to the following standards to obtain the target values: compressive strength EN 12390-3 (2003); flexural strength EN 12390-5 (2009); static modulus of elasticity EN 12390-13 (2014); dynamic modulus of elasticity EN 12504-4 (2005). Samples for testing of mechanical properties were stored and cured in a stable laboratory environment during solidification and maturation, and after 28 and 60 days were determined by laboratory tests. Compressive strength was tested on cubic specimens 150 × 150 × 150 mm^3^ and other properties were tested on prismatic specimens 100 × 100 × 400 mm^3^.

Water absorption capacity by immersion was tested on cubic specimens 100 × 100 × 100 mm^3^. Specimens were treated by water, and after stabilization of weight, dried in an oven at a temperature of 105 ± 2 °C until stabilization of weight. The saturated surface-dried density and dry density were measured on these samples. Samples NAC 1 and RMAC 1 for testing were stored and cured in a stable laboratory environment, and samples NAC 2 and RMAC 2 were stored and cured in water during solidification and maturation for 60 days to verify the influence of activation of the crystalline admixture by water. Capillary water absorption was determined by measuring the rate of water absorption by the capillaries. The ends of the fractured prismatic specimens of 100 × 100 × approx. 150 mm^3^, which were tested after the flexural strength test, were immersed in water up to a maximum height of 5 mm and measured by periodically weighing the surface-dried sample until their weight stabilized.

Prismatic specimens 100 × 100 × 400 mm^3^ for examination of thefreeze-thaw resistance were stored and cured in water during solidification and maturation for 28 and 60 days. Subsequently, the samples were placed in freezing-thawing equipment KD 20 developed by the Ecofrost company (Czech Republic, Olomouc) for testing frost resistance according to the Czech standard CSN 73 1322 (1969). Test specimens occurred in freezing cycles in which the temperature of the freezer must be between −15 °C and +20 °C. One freezing cycle consists of four hours of freezing and two hours of thawing. During freezing, the test specimens are stored in water of +20 °C. At the end of each freezing stage (25 cycles), one set of surface-dried test beams (3 pieces) shall be tested to determine their dimensions, weight, bulk density, and dynamic modulus of elasticity according to EN 12504-4 (2005). After the frost resistance test (typically 100 cycles), the test beams are tested for flexural strength according to EN 12390-5 (2009).

Prismatic specimens 100 × 100 × 400 mm^3^ for examination of the carbonation resistance of mixtures NAC 1 and RMAC 1 were stored and cured in a stable laboratory environment, and samples NAC 2 and RMAC 2 were stored and cured in water during solidification and maturation for 60 days to verify the influence of activation of the crystalline admixture by water. Afterwards, the specimens were stored in a laboratory incubator with air circulation with CO_2_ atmosphere CO2CELL (MMM group, Germany, Munich). Samples were placed in the environment with 3.0 ± 0.2% CO_2_ concentration for 28 days, which was inspired by the Standard CSN EN 12390-12. However, the evaluating of the carbonation resistance was modified to compare the influence of the same environment to the different mixtures with different amounts of the crystalline admixture. Furthermore, the flexural strength and carbonation depth were measured. The carbonation depth was evaluated on the prismatic specimens split in halves by the use of the phenolphthalein indication method, due to the reduction of pH in concrete by the effect of CO_2_. The solution of 0.8 g of phenolphthalein powder was dissolved in a solution of 70 mL ethanol and 30 mL of deionized water.

## 3. Results and Discussion

### 3.1. Physical Properties

**Figure 3:**
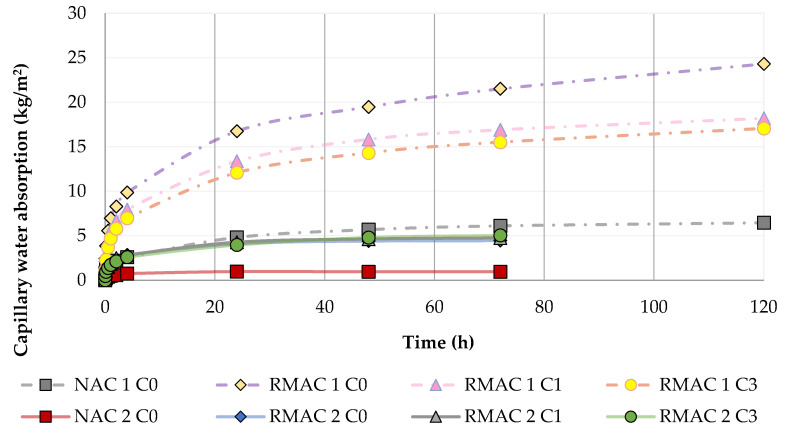
Comparison and progression of capillary water absorption of NAC and RMAC.

The water absorption of concrete influences its durability [78,79]. The values of density, water absorption by immersion, and capillary water absorption are shown in Table 3. The density of RMAC is lower in comparison with NAC, with a maximal decline of 25%. Test results of the density showed no significant relationship depending on the amount of crystalline admixture in the RMAC mixture. The water absorption by immersion was more than three times higher for the RMAC in comparison with NAC. In addition, the results of the capillary water absorption of RMAC showed higher values in comparison with NAC (see Figure 3). Furthermore, the capillary water absorption was higher for NAC 1 and RMAC 1 mixtures due to the different concrete treatment during solidification and maturation. However, both types of water absorption of RMAC 2 mixtures increased with higher amounts of crystalline admixture. On the contrary, both types of water absorptions of RMAC 1 mixtures decreased with higher amounts of crystalline admixture. This was probably caused by the different concrete treatment during solidification and maturation and shows the impact of the activating of crystalline admixture during curing in water. Examined results of the density and water absorption of RMAC mixtures confirm the results reported in previous studies [34,78,79,80,81].

### 3.2. Mechanical Properties

**Figure 4:**
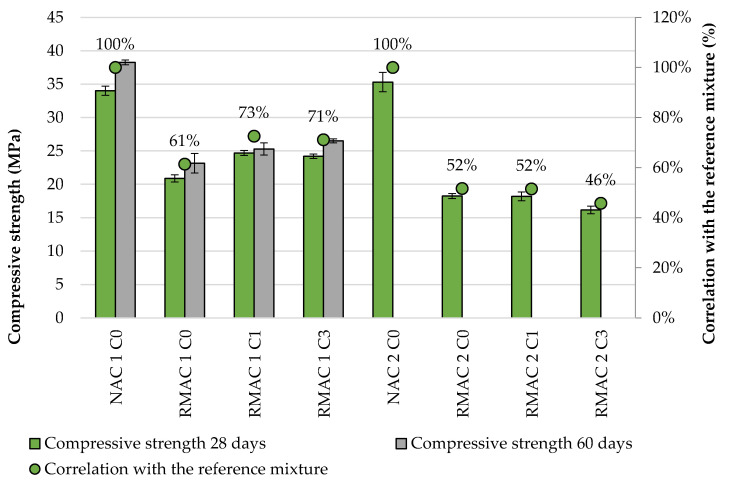
Comparison of compressive strength of NAC and RMAC at age 28 and 60 days.

The compressive and flexural strength and static and dynamic modulus of elasticity were tested for all concrete mixtures due to the knowledge of the material properties before durability examination (see Table 4). The compressive strength is one of the most important properties of concrete, which determines its use. This property is mostly negatively influenced by using recycled aggregate, especially from recycled masonry [30,31,32,33,34,35,36,37,38,39,40,41,42,43,44,45,46,47,48,49,50,51,52,53,54,55,56]. The compressive strength was evaluated on the cubic specimens before storing prismatic specimens in the freeze-thaw chamber. The testing age of samples NAC 1 and RMAC 1 was 28 days and 60 days for the samples NAC 2 and RMAC 2. The samples NAC1 and RMAC1 were also tested after 60 days for comparison with samples NAC2 and RMAC 2. Test results of the compressive strength showed lower values for all tested RMACs in comparison with the NAC, with a maximal decline of 54% (see Figure 4). There are also differences between the compressive strength of RMAC 1 and RMAC 2 mixtures, which were probably caused by the different quality of RMA. Test results also verify the increasing compressive strength with time, however, without significant influence of the crystalline admixture. The flexural strength of RMAC mixtures was also lower in comparison with NAC. The lowest decline was measured for the RMAC 2 mixture which was up to 50%, while the maximal decline of RMAC 1 mixtures was up to 30%. The addition of the crystalline admixture shows the opposite influence for RMAC 1 and RMAC 2 mixtures. As reported in previous studies [82,83,84], the static modulus of elasticity decreases more than other mechanical properties while replacing RMA in concrete mixture. This decline for full replacement rates was more than 50%, which applied in this case too. The decline of dynamic modulus of elasticity shows the highly negative influence of replacing NA in concrete mixture, with a maximal decline more than 50%. For this property, a negative impact of crystalline admixture was also shown, which decreased the dynamic modulus between 5 and 10%.

### 3.3. Durability Properties

The durability of concrete is essential due to the desire to maintain performance of concrete throughout the service life of a structure. This study deals with two durability properties in which the positive influence of the addition of crystalline admixture was predicted. The evaluated properties were freeze-thaw resistance, which is essential for future utilization in the outdoor environment, and carbonation depth, which it is necessary to know due to the reinforcement design.

#### 3.3.1. Freeze-Thaw Resistance

Freezing and thawing are often used as one possible way to evaluate concrete durability. The freeze-thaw resistance of concrete is mostly evaluated by measuring the dynamic elastic modulus, weight loss rate, and flexural strength loss rate after exposure to freeze-thaw cycles. Generally, this property is mainly affected by porosity, water content, environmental conditions, and aggregate types [13].

**Figure 5:**
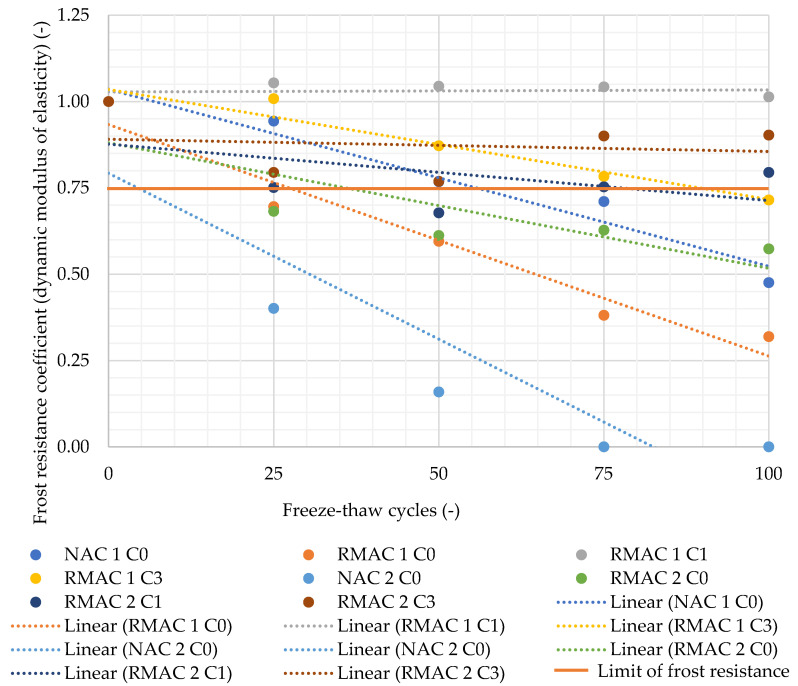
Frost resistance coefficient determined from the dynamic modulus of elasticity.

**Figure 6:**
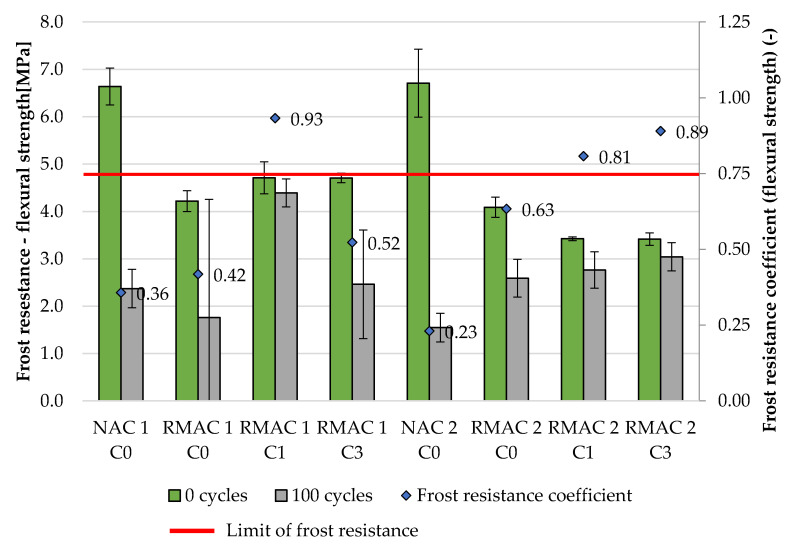
Flexural strength for 28 days (before freezing), after 100 freeze-thaw cycles and frost resistance coefficient determined from flexural strength.

Two properties were evaluated for samples exposed to freezing and thawing cycles: dynamic modulus of elasticity and flexural strength. The dynamic modulus of elasticity was evaluated after 0, 25, 50, 75, and 100 cycles (Table 5) The resulting values were compared to the reference values, which were measured on the samples after 28 days, then they were placed in freezing-thawing chamber. The frost resistance coefficient was determined from these values as the quotient of two values measured before and after freezing and thawing. The frost resistance coefficient and its linear developing trend are shown in Figure 5. The evaluation of flexural strength after 0 and 100 cycles was also performed. The frost resistance coefficient was determined from the flexural strength before and after freezing and thawing cycles the same way as for the dynamic modulus of elasticity (see Table 6 and Figure 6). Concrete mixture is frost resistant when the frost resistance coefficient does not decrease under the value 0.75.

The results of the dynamic modulus of elasticity tested during the freezing and thawing cycles by the ultrasonic method show a significant influence of crystalline admixture. All four mixtures without crystalline admixture have the lowest freeze-thaw resistance according to the frost resistance coefficient determined from the dynamic modulus of elasticity, which means that the frost resistance coefficient was lower than 0.75. Three mixtures (NAC 1, 2 and RMAC 2 C0) was not frost resistant at all. Mixture RMAC 1 C0 was frost resistant until 50 cycles. On the contrary, the majority of mixtures with crystalline admixture were frost resistant for the whole test period, which was 100 cycles. Only the mixture RMAC 1 C3 was frost resistant until 75 cycles. Furthermore, the results of freeze-thaw resistance of concrete mixtures with crystalline admixture determined from the dynamic modulus of elasticity verify the positive impact of crystalline admixture in time due to the testing age of the mixtures, when the mixtures RMAC 2 were stored in the freezing-thawing chamber at age 60 days, unlike mixtures RMAC 1, which were stored in the freezing-thawing chamber at age 28 days.

**Figure 7:**
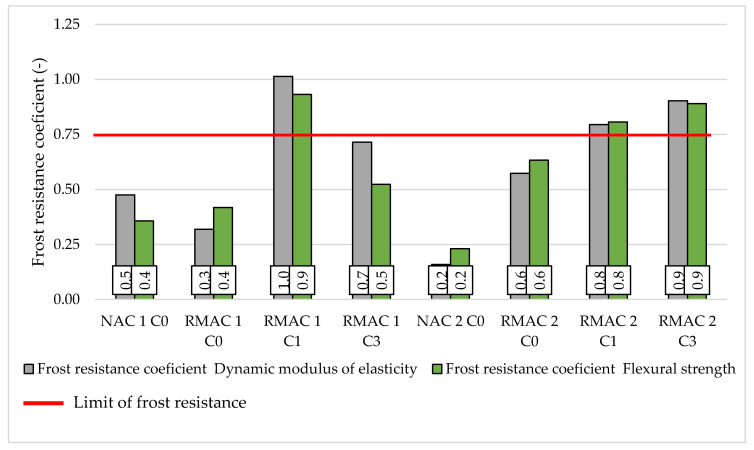
The comparison of the frost resistance coefficients evaluated from dynamic modulus of elasticity and flexural strength.

The second possibility of evaluating the frost resistance according to the Czech standard is the frost resistance coefficient determined from flexural strength. This test could be measured only on samples which were not damaged during freeze-thaw cycles. In the case of this study, the flexural strength could be tested after 100 freeze-thaw cycles for the majority of concrete mixtures, however, the mixture NAC 2 C0 was tested after only 50 cycles and for mixture RMAC 1 C0 stayed testable in only one sample after 100 cycles. In general, the results of the flexural strength correspond with the results of dynamic modulus of elasticity and show a significant influence of crystalline admixture (see Figure 7). All four mixtures without crystalline admixture have the lowest freeze-thaw resistance according to the frost resistance coefficient determined from the flexural strength, which means that the frost resistance coefficient was lower than 0.75. On the contrary, the majority of mixtures with crystalline admixture were frost resistant for 100 cycles. Only the mixture RMAC 1 C3 had a lower frost resistance coefficient than 0.75, which was 0.52. Furthermore, the results of freeze-thaw resistance of concrete mixtures with crystalline admixture determined from the flexural strength show the same positive impact of crystalline admixture in time due to the testing age of the mixtures as was described for results of frost resistance determined from dynamic modulus of elasticity.

#### 3.3.2. Carbonation Resistance

Carbonation of concrete can be described as a physicochemical process in which a number of chemical reactions take place in the presence of carbon dioxide (CO_2_), which promotes the reduction of pH in concrete. There are many studies about the positive or negative influence of CO_2_ to the microstructure and properties of concrete. On one hand, carbonation can cause important changes in porosity, pore size distribution, connectivity, specific surface and also significant changes in transport properties, including water permeability, capillary retention, ion and gas diffusion. Furthermore, the decalcification of the C-S-H phase was found [85]. On the other hand, carbon dioxide, which penetrates the concrete mainly through a diffusion mechanism, slowly progresses from the surface of the concrete. In the presence of moisture, CO_2_ forms carbonic acid, which reacts with calcium hydroxide (Ca (OH)_2_) to form calcium carbonate (CaCO_3_) and can slightly increase strength and reduce permeability due to the deposition of CaCO_3_ in the cavities of the cement matrix [9,86].

**Figure 8:**
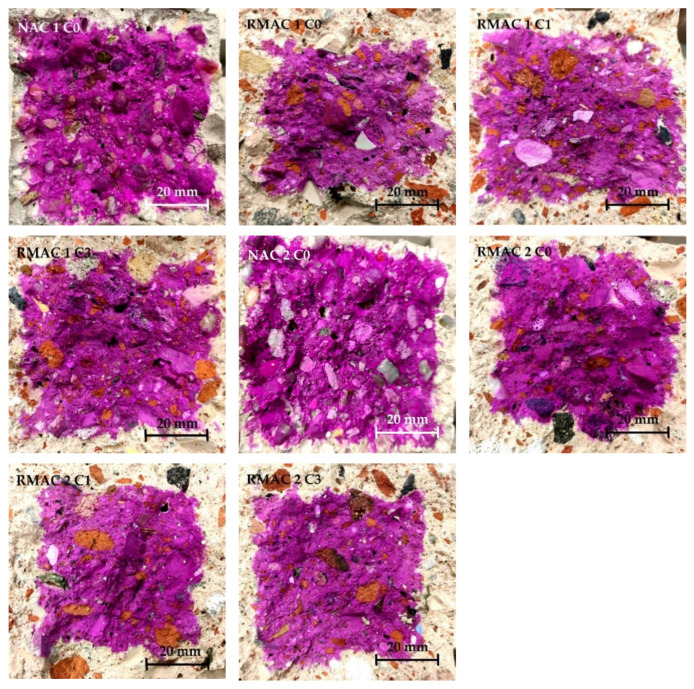
Carbonation depth of NAC and RMAC.

Three properties were evaluated for samples exposed to the high concentration of CO_2_. The depth of decrease of pH of concrete was measured by the phenolphthalein method (see Figure 8). Furthermore, the dynamic modulus of elasticity and flexural strength were evaluated. These properties were tested on the prismatic specimens which were placed in the laboratory incubator with air circulation with a CO_2_ atmosphere. The dynamic modulus of elasticity and flexural strength of concrete mixtures was also evaluated before placing the samples to the laboratory incubator with air circulation with CO_2_ atmosphere and after 28 days effecting 3.0 ± 0.2% of CO_2_ (see Table 7). The resulting values were compared to the reference values, which were measured on the samples after 28 days before then they were placed in an environment with a higher value of CO_2_.

**Figure 9:**
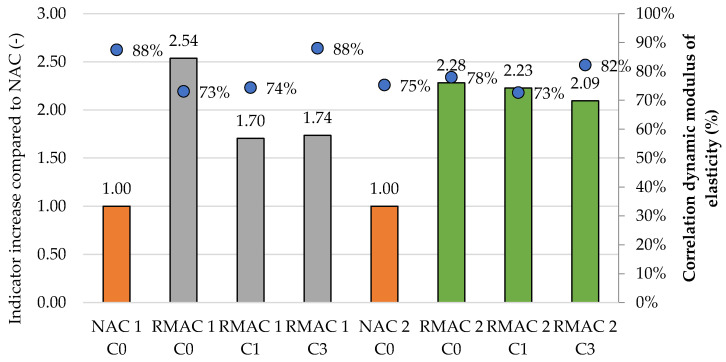
Carbonation resistance—the indicator increases of measured carbonation depth compared to NAC and correlation of dynamic modulus of elasticity of NAC and RMAC samples no exposed to CO_2_ and exposed to CO_2_.

In general, the results of the carbonation show the deep effect of CO_2_ for all RMAC mixtures, which shows no significant improvement in the carbonation resistance of RMAC with the addition of crystalline admixture. The results are evaluated by the indicator of increase the depth of carbonation compared to the NAC. This indicator of RMAC mixtures was more than twice as high as in NAC mixtures. This result slightly corresponds with the results presented in the previous study [9], where carbonation depth of RAC with 100% replacement rate of coarse aggregate was 2.5 times higher in comparison with NAC; however, the carbonation depth of RAC with the same replacement rate of fine aggregate was 8.7 times higher. In this study, both fractions of RMA were used for concrete. In addition, the indicator of RMAC mixtures without crystalline admixture is slightly higher in comparison with RMAC with crystalline admixture, which is more visible for mixture RMAC 1, in which the indicator decreases from 2.5 to approximately 1.7. On the contrary, the clearly positive effect was not shown for mixture RMAC 2. Furthermore, the influence of water curing and longer curing of concrete mixtures with crystalline admixture, where RMAC 2 mixtures were 60 days treated in water, was not shown to have a significant effect on the carbonation depth (see Figure 9).

The flexural strength of all RMAC mixtures after exposure to CO_2_ is slightly lower in comparison with the sample which was not exposed to CO_2_. However, there was no significant decline of flexural strength of RMAC 2 mixtures with the crystalline admixture, which could show the positive impact of longer water curing of samples (see Table 7). In addition, the dynamic modulus of elasticity of RMAC mixtures with crystalline admixture had also shown better results and a lower decline of properties for samples with crystalline admixture. The correlations of the dynamic modulus of elasticity before and after the exposition of CO_2_ are shown in Figure 8.

## 4. Conclusions

In this study, the experimental verification of the improvement of the physical, mechanical and durability properties of concrete containing recycled masonry aggregate by crystalline admixture was examined and discussed. It is generally known that the mechanical properties and durability decrease with the replacement of natural aggregate by recycled aggregate. The durability of recycled masonry aggregate concrete is negatively influenced by higher porosity and water absorption of concrete, which could be improved by adding suitable admixture. In the previous studies, a few possibilities of improvement of properties using admixtures have been tested. In this case, the improvement of the durability of recycled masonry aggregate concrete by the crystalline admixture was verified, which has been carried out for the first time in this study. The final conclusions that have been reached can be summarized in the following points:

1. The improvement of mineral admixture utilization was verified. However, it was found it that has no significant positive impact on the evaluated mechanical properties.
2. The water absorption by immersion was approximately three times higher without the positive effect of crystalline admixture. On the contrary, capillary water absorption verified the positive impact of crystalline admixture, however, it was still more than two times higher.
3. The utilization of crystalline admixture leads to better freeze-thaw resistance of recycled masonry aggregate concrete, which meets the requirement of frost resistance according to the Czech standard.
4. The carbonation depth of RMAC was more than two times higher in comparison with conventional concrete. For one set of samples (mixtures RMAC 1), the carbonation depth was clearly improved by the crystalline admixture.

The novelty of this study was the utilization of crystalline admixture to improve the durability of concrete. It was predicted that this admixture could fill the pores due to its reaction with water, which is contained in recycled masonry aggregate. The positive impact of crystalline admixture is not clearly shown for water absorption capacity of recycled masonry aggregate concrete due to different results of water absorption by immersion and capillary water absorption. However, there are clear benefits of crystalline admixture utilization on the durability, especially on freeze-thaw resistance. Although the laboratory measurements have been done with emphasis on different types of curing and concrete ages, the goal of further laboratory measurements will be confirmation of these results.

## Figures and Tables

**Table 1:** Physical properties of particular fractions of used aggregates.

Types of Recycled Aggregate	Grading (mm)	Content of Finest Particles	Oven-Dried Particle Density		Water Absorption Capacity (%)	
		f (%)	ρ_RD_ (kg/m^3^)	σ	WA_24_ (%)	σ
Natural aggregate (NA)	0–4	2.0	2570	81	1.0	0.0
	4–8	0.1	2530	12	1.7	0.3
	8–16	0.2	2540	12	1.9	0.2
Recycled masonry aggregate 1 (RMA 1)	0–4	2.9	2340	108	3.7	0.6
	4–8	0.4	1920	62	12.4	0.7
	8–16	0.7	2130	72	7.8	0.5
Recycled masonry aggregate 2 (RMA 2)	0–4	1.3	1950	166	13.3	2.1
	4–8	0.4	2050	41	10.6	0.4
	8–16	0.3	1990	33	10.6	1.1

**Table 2:** Concrete mix proportion, per cubic meter.

Designation	NAC 1 C0	RMAC 1 C0	RMAC 1 C1	RMAC 1 C3	NAC 2 C0	RMAC 2 C0	RMAC 2 C1	RMAC 2 C3
Cement (kg/m)	260	260	260	260	260	260	260	260
Water (kg/m^3^)	169	219	219	219	169	284	284	284
Sand (kg/m^3^)	710	0	0	0	710	0	0	0
NA 4/8 (kg/m^3^)	520	0	0	0	520	0	0	0
NA 8/16 (kg/m^3^)	609	0	0	0	609	0	0	0
RMA 0/4 (kg/m^3^)	0	807	807	807	0	949	949	949
RMA 4/8 (kg/m^3^)	0	54	54	54	0	32	32	32
RMA 8/16 (kg/m^3^)	0	653	653	653	0	500	500	500
Crystalline admixture (kg/m^3^)	0	0	5	10	0	0	5	10
w/c eff (-)	0.65	0.65	0.65	0.65	0.65	0.65	0.65	0.65
w/c (-)	0.65	1.09	1.09	1.09	0.65	0.84	0.84	0.84

**Table 3:** Average values of results of physical properties of concrete, including standard deviation.

Recycled Concrete Mixture	Dry Density		Water Absorption by Immersion		Capillary Water Absorption	
Designation	(kg/m^3^)	σ	(%)	σ	(kg/m^2^)	σ
NAC 1 C0	2240	21	5.3	0.1	6.45 ^(1)^	0.68
RMAC 1 C0	1726	22	17.3	0.7	24.31 ^(1)^	2.08
RMAC 1 C1	1765	65	17.0	1.0	18.19 ^(1)^	1.59
RMAC 1 C3	1755	26	16.6	0.3	17.05 ^(1)^	1.18
NAC 2 C0	2141	32	5.5	0.2	0.93 ^(2)^	0.25
RMAC 2 C0	1708	21	17.0	0.8	4.45 ^(2)^	0.69
RMAC 2 C1	1673	20	18.1	0.3	4.74 ^(2)^	0.61
RMAC 2 C3	1659	7	18.3	0.4	5.03 ^(2)^	0.11

^(1)^ Weight stabilization after 120 h; ^(2)^ Weight stabilization after 72 h.

**Table 4:** Average values of results of mechanical properties of concrete, including standard deviation.

Recycled Concrete Mixture	Compressive Strength				Flexural Strength		Static Modulus of Elasticity		Dynamic Modulus of Elasticity	
	28 Days		60 Days							
Designation	(MPa)	σ	(MPa)	σ	(MPa)	σ	(GPa)	σ	(GPa)	σ
NAC 1 C0	34.0	0.7	38.2	0.4	6.6	0.4	38.2	4.8	34.8	4.1
RMAC 1 C0	20.9	0.5	23.1	1.5	4.2	0.2	15.9	1.7	21.4	1.1
RMAC 1 C1	24.7	0.4	25.3	0.9	4.7	0.3	15.9	0.2	21.2	1.0
RMAC 1 C3	24.2	0.3	26.5	0.3	4.7	0.1	15.2	0.5	19.9	2.4
NAC 2 C0	-	-	35.3	1.4	6.7 ^(1)^	0.7	33.8 ^(1)^	0.4	39.1	5.1
RMAC 2 C0	-	-	18.2	0.4	4.1 ^(1)^	0.2	16.9 ^(1)^	0.7	22.0	1.4
RMAC 2 C1	-	-	18.2	0.7	3.4 ^(1)^	0.0	15.4 ^(1)^	0.1	21.9	1.5
RMAC 2 C3	-	-	16.2	0.6	3.4 ^(1)^	0.1	15.9 ^(1)^	0.4	18.9	1.0

^(1)^ Examined in 60 days.

**Table 5:** Dynamic modulus of elasticity measured by ultrasonic method and frost resistance coefficient determined from the dynamic modulus of elasticity after freezing and thawing cycles.

Recycled Concrete Mixture	Dynamic Modulus of Elasticity (GPa) + Frost Resistance Coefficient (-)									Freeze-Thaw Resistance
Designation	0 cycles	25 Cycles		50 Cycles		75 Cycles		100 Cycles		Cycles
NAC 1 C0	34.8	32.8	0.94	26.7	0.77	24.7	0.71	16.5	0.48	50
RMAC 1 C0	21.4	14.9	0.70	12.8	0.59	8.2	0.38	6.8	0.32	0
RMAC 1 C1	21.2	22.3	1.05	22.1	1.04	22.0	1.04	21.4	1.01	100
RMAC 1 C3	19.9	20.1	1.01	17.4	0.87	15.6	0.78	14.2	0.71	75
NAC 2 C0	39.1	15.7	0.40	6.2	0.16	-	-	-	-	0
RMAC 2 C0	22.0	15.0	0.68	13.5	0.61	13.8	0.63	12.6	0.57	0
RMAC 2 C1	21.9	16.4	0.75	14.8	0.68	16.5	0.75	17.4	0.79	100
RMAC 2 C3	18.9	15.0	0.79	14.5	0.77	17.0	0.90	17.1	0.90	100

**Table 6:** Flexural strength and frost resistance coefficient determined from flexural strength after freezing and thawing cycles.

Recycled Concrete Mixture	Flexural Strength				Frost Resistance Coefficient
	(MPa)		σ		(-)
Designation	0	100	0	100	
NAC 1 C0	6.6	2.4	0.4	0.4	0.36
RMAC 1 C0	4.2	1.8	0.2	2.5	0.42
RMAC 1 C1	4.7	4.4	0.3	0.3	0.93
RMAC 1 C3	4.7	2.5	0.1	1.1	0.52
NAC 2 C0	6.7	1.5	0.7	0.3	0.23
RMAC 2 C0	4.1	2.6	0.2	0.4	0.63
RMAC 2 C1	3.4	2.8	0.0	0.4	0.81
RMAC 2 C3	3.4	3.0	0.1	0.3	0.89

**Table 7:** Flexural strength, dynamic modulus of elasticity and carbonation depth after 28 days in laboratory incubator with air circulation with CO_2_ atmosphere.

Recycled Concrete Mixture	Flexural Strength				Dynamic Modulus of Elasticity				Indicator of Increase of Carbonation Depth Compared to NAC
	No Exposition to CO_2_		Exposition to CO_2_		No Exposition to CO_2_		Exposition to CO_2_		
Designation	(MPa)	σ	(MPa)	σ	(GPa)	σ	(GPa)	σ	(mm)
NAC 1 C0	6.6	0.4	6.4	0.6	34.8	4.1	30.5	0.7	1.00
RMAC 1 C0	4.2	0.2	3.9	0.1	21.4	1.1	15.7	0.8	2.54
RMAC 1 C1	4.7	0.3	4.0	0.2	21.2	1.0	15.8	0.9	1.70
RMAC 1 C3	4.7	0.1	4.1	0.2	19.9	2.4	17.5	0.9	1.74
NAC 2 C0	6.7	0.7	7.4	0.4	39.1	5.1	29.4	4.2	1.00
RMAC 2 C0	4.1	0.2	3.6	0.3	22.0	1.4	17.2	1.0	2.28
RMAC 2 C1	3.4	0.0	3.4	0.2	21.9	1.5	15.9	0.9	2.23
RMAC 2 C3	3.4	0.1	3.4	0.1	18.9	1.0	15.5	1.4	2.09
